# Circulatory Estrogen Level Protects Against Breast Cancer in Obese Women

**DOI:** 10.2174/1574892811308020004

**Published:** 2013-05

**Authors:** Zsuzsanna Suba

**Affiliations:** National Institute of Oncology, Budapest, Hungary

**Keywords:** Androgen, breast cancer risk, estrogen, insulin resistance, menopause, metabolic syndrome, obesity, type-2 diabetes, visceral adiposity.

## Abstract

Literary data suggest apparently ambiguous interaction between menopausal status and obesity-associated breast cancer risk based on the principle of the carcinogenic capacity of estrogen. Before menopause, breast cancer incidence is relatively low and adiposity is erroneously regarded as a protective factor against this tumor conferred by the obesity associated defective estrogen-synthesis. By contrast, in postmenopausal cases, obesity presents a strong risk factor for breast cancer being mistakenly attributed to the presumed excessive estrogen-production of their adipose-tissue mass. Obesity is associated with dysmetabolism and endangers the healthy equilibrium of sexual hormone-production and regular menstrual cycles in women, which are the prerequisites not only for reproductive capacity but also for somatic health. At the same time, literary data support that anovulatory infertility is a very strong risk for breast cancer in young women either with or without obesity. In the majority of premenopausal women, obesity associated insulin resistance is moderate and may be counteracted by their preserved circulatory estrogen level. Consequently, it is not obesity but rather the still sufficient estrogen-level, which may be protective against breast cancer in young adult females. In obese older women, never using hormone replacement therapy (HRT) the breast cancer risk is high, which is associated with their continuous estrogen loss and increasing insulin-resistance. By contrast, obese postmenopausal women using HRT, have a decreased risk for breast cancer as the protective effect of estrogen-substitution may counteract to their obesity associated systemic alterations. The revealed inverse correlation between circulatory estrogen-level and breast cancer risk in obese women should advance our understanding of breast cancer etiology and promotes primary prevention measures. New patents recommend various methods for the prevention and treatment of obesity-related systemic disorders and the associated breast cancer.

## INTRODUCTION

Many studies support an apparently Janus-faced ambiguous interaction between obesity and breast cancer risk depending on the menopausal status of patients [1-3]. In young women, before menopause adiposity is erroneously regarded to exhibit a protective effect against breast cancer risk [1, 4, 5]. By contrast, in postmenopausal cases, particularly in the elderly, the association is highly positive, obesity confers a strong risk for breast cancer [2, 6]. Controversial results of clinical studies mistakenly suggest that obesity is advantageous against breast cancer by the defective estrogen synthesis in young women [1]. This assumption seems to be consistent with an inverse relationship between the BMI and serum estradiol levels found in premenopausal cases, particularly in the follicular phase of the cycle [7]. Conversely, postmenopausal obesity is harmful by the erroneously presumed excessive estrogen production of adipose tissue mass in older women [8-10]. Explanations for these supposed ambiguous correlations are in concordance with the preconception that high estrogen levels, either endogenous or exogenous, play crucial role in mammary carcinogenesis [11].

Nevertheless, several authors could find confusing and disturbing associations between obesity and breast cancer risk in postmenopausal women. Obese postmenopausal women, who had never used hormone replacement therapy (HRT) exhibited fairly high breast cancer risk [12]. By contrast, HRT use attenuates or abolishes the increased breast cancer incidence [13-15] suggesting a protective impact of female sexual hormones in aged obese women.

In premenopausal cases, the results of clinical studies justify that obesity induces mild or moderate decrease in circulating estrogen levels reflected by their inclination to anovulatory infertility and long or irregular menstrual cycles [16]. Based on the traditional concept of estrogen induced mammary carcinogenesis, obesity associated defective estrogen production in premenopausal women seems apparently to justify the breast cancer preventive impact of fatness.

Even larger studies, which equivocally strengthen the protective effect of obesity for premenopausal breast cancer risk, confess that the responsible mechanisms are completely obscure. Evidences, provided by clinical endocrinological studies regarding correlations between defective hormonal status of obese women and decreased breast cancer incidence, are inconsistent or fairly contradictory [1]. Considering the whole spectrum of unexplained, apparently ambiguous biological behavior as regards breast cancers arising in premenopausal and postmenopausal women, the existence of two distinct types of breast malignancies was presumed occurring before and after menopause [17, 18].

Obesity is a well-known cancer risk factor associated with different grades of insulin resistance and a disturbance of male to female circulating sexual steroid levels [19, 20]. Hyperinsulinemia, dyslipidemia, elevated fasting glucose level and type-2 diabetes are the well documented concomitants of adiposity associated insulin resistance [21]. Obesity provokes further alterations in the endocrine system as well conferred by an excessive circulatory androgen level at the expense of defective estrogen synthesis [22]. Nevertheless, the health benefit of pathologic states; such as overweight and obesity can hardly be justified at any age of women.

The aim of the present study is to clarify the realistic correlations between breast cancer risk and obesity-associated hormonal alterations during the whole life of women. Moreover, this study tries to reveal the sources of the misleading clinical and epidemiologic results suggesting the breast cancer protective effect of obesity in the young. These associations are examined in an analytical review based on the results of prospective, case-control and meta-analytic studies.

### Pathogenetic Mechanisms as Links Between Obesity and Breast Cancer Risk

**Fig. (1):**
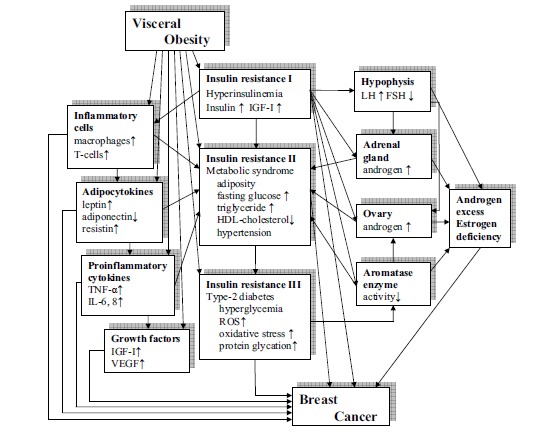
Pathogenetic mechanisms of obesity-related risk for breast cancer.

Clinical and experimental evidences prove that obesity, particularly visceral fatty tissue deposition leads to insulin resistance, associated with diverse immunologic, metabolic and hormonal alterations mediating breast cancer risk (Fig. **1**). The main stream of obesity related alterations is a self-generating, progressive insulin resistance in thorough interplay with the dysmetabolism and inflammation of adipose tissue mass and with an imbalance of sexual steroid production in the endocrine organs.

### Obesity Related Insulin Resistant States

Insulin resistance (IR) is a defect of insulin-mediated cellular glucose uptake, which may elicit many disorders in the gene regulation of cellular metabolism, growth, differentiation and mitotic activity. The progression of this disorder predisposes patients to a variety of diseases, such as metabolic syndrome, type-2 diabetes, cardiovascular lesions and malignancies at different sites [23].

#### Reactive hyperinsulinemia

in the first, compensated phase of insulin resistance maintains serum glucose level within the normal range by means of an increased secretory capacity of insular β-cells [24]. Insulin has diverse metabolic effects and at the same time functions as a growth factor with strong mitogenic capacity. High insulin level in itself may be regarded as cancer risk by the excessive stimulation of multiple cellular signaling cascades [25]. Hyperinsulinemia increases the hepatic synthesis and mitogenic activity of other, insulin-like growth factors, such as IGF-I. High levels of insulin and IGF-I have important role in the alterations of cell proliferation and in tumor induction, particularly in breast and prostate [26]. In a case-cohort study insulin and IGF-I levels were positively associated with breast cancer risk suggesting that hyperinsulinemia may be a substantial link between obesity and mammary carcinogenesis [27]. A recent patent introduced somatostatin analogues and IGF-I inhibition for the prevention and treatment of preneoplastic lesions and cancers of the breast [28].

#### Metabolic syndrome (prediabetes)

develops in the second, partially uncompensated phase of insulin resistance when the enhanced insulin synthesis of pancreatic islet cells is not enough to maintain euglycemia. This is a quartet of elevated fasting glucose, high serum triglyceride, low HDL cholesterol level and hypertension, being characteristic of viscerally obese patients [29]. Each of these symptoms alone is risk factor for cancer, and together they mean a multiple risk [30, 31].

*Hyperglycemia* is advantageous for the unrestrained DNA synthesis of tumor cells. It provokes deliberation of free radicals causing derangements in both DNA and enzymes having important role in repair mechanisms [32, 33]. High serum glucose level leads to a non-enzymatic glycation of protein structures, and the glycated products enhance the deliberation of free radicals, cytokines and growth factors [34]. A prospective study on the role of glucose metabolism in breast cancer incidence justified that hyperglycemia among insulin resistant women exhibits direct correlation with mammary cancer risk [35].

*Dyslipidemia* is a complex disturbance of the lipid metabolism associated with insulin resistance and hyperinsulinemia [36]. Serum level of triglycerides shows close parallelism with insulin resistance, serum insulin level and BMI, whereas being inversely correlated with the high density lipoprotein (HDL) level. Correlation of dyslipidemia and malignant tumors was thoroughly studied in colorectal and breast cancer cases [37, 38]. Results of clinical examinations justified the close association between hypertriglyceridemia and breast cancer [39].

*Hypertension* usually shows close positive correlation with obesity, insulin resistance and hyperinsulinemia [40, 41]. Elevated insulin level is closely associated with increased activity of the sympathetic nervous system resulting in adrenergic vasoconstriction and hypertension [24, 42]. There are further crossroads between obesity and hypertension by means of hormonal regulation. Activation of the renin-angiotensin-aldosterone system and subsequent elevations in angiotensin II and aldosterone levels are frequently seen in metabolic syndrome [43]. In postmenopausal hypertension, changes in androgen/estrogen ratios favoring for androgens have been implicated to play an important causal role. After menopause, hypertension proved to be a strong risk factor for hormone dependent tumors, separately from obesity [44].

Metabolic syndrome is associated with both increased breast cancer risk and high mortality rates in postmenopausal cases [45, 46]. Time-dependent covariate analyses indicated a positive association between the metabolic syndrome and breast cancer, due primarily to positive associations with serum glucose, serum triglycerides, and diastolic blood pressure [47].

#### Type-2 diabetes

is the uncompensated phase of insulin resistance when the secretory capacity of insular β-cells becomes exhausted and the decreased serum insulin level results in hyperglycemia. The disrupted glucose homeostasis, the excessive formation of free radicals and the protein glycation depress the activities of the antioxidant scavengers and enzymes. These noxious processes cause serious damages in all biological structures even at a molecular level [32, 48]. The role of free radicals and oxidative stress in the process of carcinogenesis is a well-known fact [49, 50]. Type-2 diabetes seems to be an independent risk for cancer development [51], particularly for breast cancer [39, 52, 53]. A recent patent reports on the invention of a pharmaceutical combination of an insulin sensitivity enhancer and a phosphoinositide 3-kinase inhibitor for the treatment of solid tumors, including breast cancer [54].

### Obesity Related Alterations in the Sexual Steroid Production

Obesity related insulin resistance and excessive insulin synthesis in women may contribute to ***hyperandrogenism*** and anovulatory infertility through several pathways as insulin is a potent regulator of sexual steroid production in the endocrine organs [55]. Hyperinsulinemia and excessive IGF-I supply stimulates *ovarian androgen production* at the expense of reduced estrogen synthesis [56]. High insulin level was found to increase the testosterone biosynthesis of human ovarian thecal cells deriving from insulin resistant, infertile women with polycystic ovarian syndrome (PCOS) [57]. Therapeutic improvement of insulin sensitivity and effective reduction of insulin secretion decreased ovarian cytochrome P450c17 alpha activity and normalized serum free testosterone level [58, 59]. A recent patent introduced a new metformin derivative as a therapeutic agent for treating metabolic syndrome, type-2 diabetes, PCOS and cancers depleted of gene P53 [60].

Hyperinsulinemia promotes *adrenal androgen synthesis* as well by means of increased adrenal sensitivity to adrenocorticotropin. At the same time, high insulin level may favor the *luteinizing hormone* *(LH) secretion of pituitary*. Increased LH level also stimulates androgen biosynthesis by the activation of adrenal gland and ovarian theca cells [61, 62]. According to earlier hypotheses, breast cancer risk is increased not only in hyperandrogenic postmenopausal women, but also in premenopausal cases with mild hyperandrogenism and apparently normal (ovulatory) menstrual cycles [63]. In centrally obese, either premenopausal or postmenopausal women, excessive ovarian production of testosterone seems to be a genetically determined risk for breast cancer [64, 65].

Insulin resistance, hyperinsulinemia and excessive IGF-I activity mediate defective estrogen synthesis by counteraction to aromatase enzyme gene expression at cellular level. The aromatase enzyme complex catalyzes the conversion of androgens to estrogens in a wide variety of tissues. In premenopausal women, the ovaries are the principle sources of estradiol; by contrast, in postmenopausal women when the ovaries cease to produce estrogen, it is synthesized in a number of extragonadal sites [66]. These sites include the adipose tissue, breast, endometrium, bone, endothelium, aortic smooth muscle cells, and numerous locations in the brain. All these tissues, particularly breast and endometrium, have high estrogen demand and a local hormone synthesis helps to preserve their structural integrity and functional activity in spite of low circulatory estrogen levels [67].

In premenopausal insulin resistant women with type-2 diabetes, the ovaries exhibit decreased capacity to convert androgen to estrogen, probably due to a reduction of ovarian aromatase activity [68]. Conversely, in patients with either estrogen deficiency (aromatase deficiency) or estrogen resistance (estrogen receptor mutation) glucose intolerance, hyperinsulinemia and lipid abnormalities are concomittant alterations associated with excessive gonadotropin and androgen levels [69].

Estrogens decrease low grade inflammatory reactions and may in parallel reduce the glucocorticoid responses. Low estrogen levels after menopause allow the predominance of glucocorticoids and the manifestation of the metabolic syndrome. These observations suggest that the disturbed equilibrium between sex hormones and glucocorticoids may be a critical element in the manifestation of metabolic syndrome-related pathologies [70].

### Inflammatory and Metabolic Dysfunctions of Adipose Tissue in Visceral Obesity

Visceral adipose tissue has important functions in energy homeostasis, metabolic equilibrium, immune responses and in the regulation of cell proliferation. Secretion of signaling molecules; adipokines regulates the cellular microenvironment both locally and systemically [71-73].

The chronic low grade inflammation associated with obesity is an important player in tumor development and progression. Increased IGF-I level may mediate the inflammation of adipose tissue via its effects on immunologically active cells including macrophages and T cells [74]. IGF-I may provoke macrophage migration and invasion and increased production of proinflammatory cytokines.

Adipokines include unique products of fatty tissue known as *adipocytokines*; such as leptin, adiponectin resistin, ghrelin etc. [75]. Leptin biosynthesis is in close direct correlation with insulin level and this may explain the increased leptin levels observed in obesity [76]. High leptin concentrations may constitute a possible link relating obesity and breast cancer promoting the invasion and migration of tumor cells [77]. Conversely, obesity may downregulate the secretion of adiponectin, an adipokine with anti-inflammatory, insulin sensitizing and anti-tumor properties [78]. The balance of leptin as well as the adiponectin concentrations are the critical factors in breast cancer risk and in other obesity related cancer genesis [79].

Proinflammatory cytokines (TNF-α, IL-6, IL-8) produced in excessive adipose tissue mass increase nitric oxide (NO) production, a substrate for reactive oxygen species (ROS). Accumulation of cytokines and ROS further contribute to insulin resistance resulting in elevated circulating glucose and free fatty acid levels. All signaling molecules of fatty tissue including cytokines, hormones and growth factors are involved in the regulation of the proliferation, invasion and metastatic spread of tumor cells [80]. Proinflammatory cytokines within the tumor microenvironment correlate with growth, increased invasiveness and poor prognosis in many types of cancer, including breast cancer [81].

### Protective Effects of Estrogen Against Obesity and Obesity Associated Alterations

Out of several hormones affecting body mass and adipose tissue deposition, estrogens promote, maintain and control the ideal distribution of body fat [82]. These steroids are known to regulate differentiation and metabolism of adipocytes as well. Estrogen deficiency and defective estrogen signaling results in obesity with increasing adipose tissue mass, preferentially in visceral location [19, 82].

### Anti-Obesity Effects of Estrogen

Estrogen regulates the metabolism, differentiation, growth and cell kinetic mechanisms of adipocytes. In healthy premenopausal women central adipocytes show higher insulin sensitivity and exhibit a higher turnover rate despite similar fat content as compared with male cases [83]. Conversely, after menopause, decreased ovarian estrogen synthesis results in increasing insulin resistance in central adipocytes and higher fasting insulin levels conferring increased risk for metabolic and cardiovascular diseases [84, 85]. In experiments, estradiol is able to inhibit the glucocorticoid production in rodent adipocytes of mesenteric origin, providing novel insight into the anti-obesity mechanism of estrogen effect [86].

Obesity and overweight are important concomitants of insulin resistance and they are strongly associated with disturbed equilibrium of male to female sexual hormone concentrations in women [87]. Healthy estrogen predominance in women induces subcutaneous gluteofemoral adipose tissue deposition [84]. Conversely, androgen excess and deficient estrogen synthesis exhibits close correlation with visceral obesity both in pre- and postmenopausal women [22]. In young women with PCOS, there are several advantages of treatment with oral contraceptives, including protection from the development of endometrial carcinoma, regularization of menses, amelioration of hirsutism, acne and obesity [88]. Similarly, in obese postmenopausal women with or without type-2 diabetes, HRT reduces abdominal adiposity and insulin resistance [89] and decreases the risk for obesity associated breast cancer [15].

### Anti-Atherogenic and Anti-Hypertensive Effects of Estrogen

Healthy premenopausal women are typically protected from cardiovascular diseases and hypertension as compared with men and this has been hypothesized to be because of the protective effects of estrogens. Conversely, obese, diabetic young women, and postmenopausal cases may lose this protected state as their bioavailable estradiol levels are strongly reduced [90].

Estrogen may have crucial role in the maintenance of normal serum lipid levels. Postmenopausal women have higher total cholesterol, LDL cholesterol and triglyceride levels, whereas lower HDL cholesterol levels as compared with premenopausal cases. These changes may be regarded as a shift toward a more atherogenic lipid profile in estrogen deficient milieu [91]. Postmenopausal estrogen therapy may reduce the risk of cardiovascular disease and this beneficial effect may be mediated in part by favorable changes in plasma lipid levels [92]. Estrogens have important regulatory role in the hepatic lipid metabolism as well. In aged rats, estradiol administration lowered the level of lipid peroxidation and improved the dysfunction parameters of the liver [93].

Estradiol has cardiovascular protective impact by its anti-hypertensive activity as well. Estrogens can downregulate components of the renin-angiotensin system (RAS) and reduce the expression and activity of angiotensin I-converting enzyme [94, 95]. Estradiol inhibits the excessive synthesis of vasoconstrictor endothelin and improves endothelial dysfunction in ovariectomized female spontaneously hypertensive rats [96]. In the pathogenesis of postmenopausal hypertension increased androgen to estrogen ratio may be associated with the activation of renin-angiotensin and endothelin systems [97].

Clinical and experimental studies support that estradiol has also antioxidant activities [98, 99] that may be effective against inflammatory lesions, atherosclerotic vessel injuries and malignancies.

### Antidiabetogenic Impacts of Estrogen

Estrogens have beneficial effects on the energy metabolism and glucose homeostasis by means of several pathways [100]. Estrogens advantageously regulate the insulin production capacity of the *pancreatic islet cells* [85]. Estrogen receptor alpha (ER-α) activation promotes β-cell mass survival and insulin biosynthesis in diabetic and obese cases, whereas ERβ activation improves glucose stimulated insulin secretion [101]. Estrogen administration seem to be a therapeutic avenue to preserve functional β-cell mass in patients with diabetes mellitus.

*In the liver*, estrogen regulates insulin sensitivity by the balanced activation of glycogen synthase and glycolytic enzymes to maintain the equilibrium of glycogen synthesis and glycogenolysis. In ER-α knockout mice, hepatic insulin resistance was associated with decreased glucose uptake in skeletal muscles [102].

*In the peripheral tissues,* ERs advantageously modulate the insulin stimulated glucose uptake through regulation of the phosphorylation of insulin receptor protein. In hyperinsulinemia, high concentrations of estradiol can inhibit the excessive insulin signaling in adipocytes [103], which may be a safety impact both against pathological glucose uptake and dangerous mitogenic activity.

ERs have crucial roles in cellular glucose uptake by regulation of intracellular glucose transporters (GLUTs) and enhancing both GLUT4 expression and translocation [104]. In human adipocytes GLUT4 abundance is highly correlated with insulin responsiveness. In women with PCOS, decrease in insulin stimulated glucose uptake was associated with reduced amount of GLUT4 on adipocyte membrane [105].

Estrogens and ER signals have pivotal role in the regulation of growth hormone (GH) activity by means of modulation of its secretion and cellular GH receptor function. Estrogens play a major and positive role in the regulation of GH-IGF-I axis in both genders [106], which may be in close correlation with their antidiabetogenic and anticancer capacities.

Postmenopausal hormone replacement therapy (HRT) reduced abdominal obesity, insulin resistance, new-onset diabetes, hyperlipidemia, blood pressure, adhesion molecules and procoagulant factors in women without diabetes and reduced insulin resistance and fasting glucose in women with diabetes [89].

### Regulation of Adipokine Secretion, Inflammatory Reactions and Growth Factor Activity by Estrogen

Low grade systemic inflammation may be associated with estrogen deficient states, namely menopause and ovariectomy. At early stages of estrogen deficiency estrogen administration decreased the inflammation associated risk of developing cardiovascular disease [107]. Nevertheless, estradiol may contribute even to the vascular healing process and to the prevention of lumen restenosis in atherogenic arteries. It improves reendothelialization through ER-α activation and decreases smooth muscle cell migration and proliferation through ER-β stimulation [108].

Phytoestrogens may advantageously influence the level of adipokines in insulin resistant women. In postmenopausal cases, diet, physical exercise and daily oral intake of soy isoflavones had a beneficial lowering effect on serum leptin, and TNF-α levels and showed a significant increase in mean serum levels of adiponectin [109]. Epidemiologic studies support that phytoestrogen rich foods may be beneficial consumed before or during adolescence for the prevention of breast cancer [110].

Estrogen advantageously regulates serum levels of available growth factors. In clinical studies estradiol lowered, whereas testosteron increased total IGF-I level and estradiol specifically suppressed unbound, free IGF-I level [111]. Crosstalk between estrogen receptors and growth factor (IGF-I, EGF, VEGF) receptor signaling pathways is well-known both in healthy tissues and malignancies [112, 113]. Nevertheless, estradiol may induce both growth stimulation and growth inhibition depending on the ratio and activity of ERs and GFRs [114].

The presumed synergistic contribution of ERs and GFRs to cancer development and progression would be a permanent danger without contraregulatory impact. Inhibition of growth factor signaling in apparently ER-negative breast cancer cells successfully restored ER expression suggesting a dynamic, inverse relationship between the two receptor systems [115]. Moreover, excessive EGF predominance decreased both ER-α protein concentration and gene transcription activity in the human breast cancer cell line MCF-7 [116]. These results suggest rather an alternative role of estrogen and growth factor actions in tumor cell proliferation.

### Correlations Between Grade and Distribution of Adiposity and Breast Cancer Risk

Fat deposition is thoroughly affected by male to female sexual steroid level [84]. Circulating estrogen level may be the key regulator in mediating differences in adipose tissue distribution between pre- and postmenopausal women [117]. In *obese premenopausal women*, peripheral, subcutaneous adiposity is typical and there is a lower incidence of obesity associated dysmetabolism. By contrast, in *obese* *postmenopausal cases*, circulating levels of estrogen are dramatically decreased and adipose tissue distribution becomes more male-like. Predominance of visceral adiposity and the associated metabolic and hormonal disorders mean high risk for obesity related diseases, included breast cancer [71].

The most important data characterizing the grade of obesity are body mass index (BMI) and weight in kilogram [4, 5, 118] reflecting general adiposity. Knowing the hormonal and metabolic background of the diverse distribution of adipose tissue, BMI or body weight in kilogram may not correctly reflect the correlations among obesity, hormonal disturbances and breast cancer risk in young women [14]. Body circumference measurements; such as hip (HC), waist (WC) and waist to hip ratio (WHR) inform about the regional distribution of fatty tissue deposition [119-121] so as to quantify the mass of visceral abdominal fat and the metabolic risk of patients.

Some authors reported on the value of WC, HC and WHC ratio in the prediction of premenopausal breast cancer occurrence [119, 122]. Conversely, in other studies, body fat distribution, such as WC had no defining role in the establishment of insulin resistance or premenopausal breast cancer risk [2, 119].

Magnetic resonance imaging (MRI) was used to quantify separately the mass of visceral and subcutaneous abdominal fat depositions in obese adolescent girls [123]. Mass of visceral fat was highly correlated with insulin secretion and insulin resistance. Conversely, abdominal subcutaneous fat mass measured by MRI did not show close correlation with the quantified indicators of insulin resistance.

In conclusion, neither BMI nor circumference measurement may exactly separate the metabolically indifferent, subcutaneous fat and the dangerous visceral fat, which may partially explain the controversial correlations between obesity grade and breast cancer risk.

### Lifelong Changes in the Estrogen Level of Women and their Relation to Obesity and Breast Cancer Risk

Circulating female sexual steroid levels and fertility continuously decrease during the life of women. The ability to conceive is at its peak in young women under 30 years of age with a continuous decline from the fourth decade, which suggests decreasing fertility even in the premenopausal phase [124]. Menopause at 50-51 years of age means a sudden break in ovarian estrogen synthesis and confers further decline in the circulating hormone level.

In premenopausal women, the good equilibrium of sex steroid synthesis defines health and reproductive capacity, whereas good, symptom-free adaptation to the estrogen deficient environment is a prerequisite of postmenopausal health. Changes in the hormonal equilibrium during women’s lives strongly influence the obesity associated breast cancer risk (Table **1**).

### Obesity Associated Hormonal Alterations in Childhood and Adolescence and their Prediction for Breast Cancer Risk

Obesity is a detrimental disorder and may not be protective against malignancies even in children. *Childhood obesity* is associated with insulin resistance and hyperinsulinemia mediating risks for chronic diseases and cancer in the adult life [125]. Many factors of metabolic syndrome, such as elevated glucose level, hypertension, high triglyceride and low HDL-cholesterol level even type-2 diabetes might occur in these young prepubertal obese cases [126].

Some studies suppose a definite key age; for example 10 years when breast cancer “protective” obesity turns to harmful between the ages of 10 and 20 years as a prediction of elevated breast cancer risk in the premenopausal life [127]. Others found fatness in childhood to be associated with decreased breast cancer risk both for pre- and postmenopausal women [128]. Further authors postulate that only teenager obesity is protective against premenopausal breast cancer initiation but if it is persistent after teenage years than it may confer an increased risk for postmenopausal breast cancer [129]. Nevertheless, finding a key age at which obesity in young girls turns from a cancer protective to a cancer promoting agent seemed to be unsuccessful.

*Obesity in puberty* with extreme somatic growth and explosion-like sexual development means a great challenge for the entire metabolic and hormonal systems. In obese children, puberty becomes a more serious danger for insulin resistant states as compared with normal weight cases [130, 131]. In obese adolescent patients, increased insulin resistance does not return to prepubertal values at the end of puberty and represents high risk for adult metabolic syndrome, type-2 diabetes and for their complications [126, 132].

In adolescent girls, obesity associated metabolic storms are related to abnormal ovarian sexual steroidgenesis as well, resulting in excessive androgen and defective estrogen production and greater frequency of irregular, anovulatory cycles [129, 133, 134]. High serum androgen concentrations developing in puberty are preserved into adulthood and are reflected by defective fertility patterns at least until 30 years of age [133, 134]. This observation may be in concordance with an increased premenopausal breast cancer risk of delayed first childbearing [18, 135].

Equilibrium of sexual hormone production and the development of regular cycles are prerequisites not only for reproduction but also for somatic health in women [19, 20, 136]. Pathological alterations in the critical period of puberty, such as obesity endangering both the fertility and survival of women might not be protective against cancer initiation in either pre- or postmenopausal cases. In conclusion, obesity related endocrine alterations in children, adolescents and at young age might really be defining factors for breast cancer risk, being not protective, but rather highly dangerous.

### Controversial Associations Between Obesity Related Anovulatory, Irregular Menstrual Cycles and Breast Cancer Risk in Premenopausal Women

In premenopausal cases, obesity is associated with defective estrogen synthesis and decreased circulatory estrogen levels, particularly in the follicular phase [7]. These obesity-related hormonal alterations may usually confer irregular menstrual cycles, anovulation, infertility and reduced response to fertility treatment [16]. Theory of estrogen induced mammary carcinogenesis supports the hypothesis that in obese young women, anovulation and the associated menstrual cycle disorders may be protective against breast cancer risk [137].

Results of cohort and case-control studies exhibit inconsistent data concerning the associations of menstrual disorders and breast cancer incidence. Some investigators found an increased breast cancer incidence in women with long menstrual cycles [138, 139] others found no associations [140-142] or conversely, a decreased risk [137, 143, 144]. In a large study, the results were quite controversial, in women having long cycles the risk of breast cancer was increased, whereas, in those whose cycle was estimated to be irregular, the risk was reduced [145]! In a prospective study, anovulatory infertility was associated with decreased breast cancer incidence [146]. By contrast, a further study of the same working group in the same year established that other factors than anovulatory disorders may be protective against breast cancer risk in obese young women [116].

Ambiguous correlations between obesity related hormonal disturbances and breast cancer risk may be explained by the fact that anovulation and menstrual irregularities are strong risk factors for breast cancer both in obese cases and non-obese controls. Among symptom-free lean control girls, laboratory findings of hyperinsulinemia and excessive androgen concentration may reveal the insulin resistance and occult anovulatory cycles [23].

In premenopausal women with polycystic ovarian syndrome (PCOS) the coexistence of anovulatory infertility and insulin resistance represents common risk for the cancers of highly hormone dependent breast, endometrium and ovary [147, 148]. Mortality data of infertile women with PCOS were examined in a long-term follow up study in the UK and breast cancer was the most common cause of death among these patients [149]. In a Brazilian study, anovulatory disorders with or without PCOS were promoters of endometrial and breast cancer by hyperinsulinemia and hyperandrogenism in infertile women [150]. In an American study, nulliparity, irregular menstrual cycles, obesity, diabetes and hypertension were strongly associated with endometrial cancer risk in premenopausal women [151]. In young, nulliparous women, endometrial carcinomas are frequently associated with synchronous primary cancers of the ovary or breast [151, 152].

Hyperprolactinemia is also associated with cycle disorders, reproductive dysfunction and hyperandrogenism in women, however, it may be sharply discerned from PCOS and other disorders related to androgen excess. An increased overall cancer risk was found in hyperprolactinemia patients but further investigations are necessary to confirm these results [153]. Data of recent studies suggest positive association between elevated prolactin level and breast cancer risk, predominantly among young, premenopausal women [154].

How can we explain the increased prevalence of breast, endometrial and ovarian cancers in women with fertility disorders, even without obesity? In premenopausal cases, slight or moderate decrease in circulating female sexual steroid levels may be enough to block the delicate mechanism of ovulation. At the same time, a slightly estrogen deficient milieu confers preferential cancer risk for the female organ triad having high estrogen demand [19, 136].

### Correlations Between Reproductive Data and Obesity-Associated Breast Cancer Risk in Premenopausal Women

In women, multiparity and risk for malignancies at several sites exhibit an inverse relationship [155-157]. High parity shows tumor protective effect even against female breast, endometrial and ovarian cancers [158, 159]. This may be attributed to the good equilibrium of female sexual steroid production associated with good fertility. Moreover, pregnancy equivalent, high female sexual hormone treatment could prevent the chemically induced mammary carcinogenesis in female Lewis rats [160].

Nulliparity may be associated with ovulatory disorders in the majority of cases and there are studies, which equivocally exhibited increased prevalence of breast cancer in nulliparous women [161, 162]. Furthermore, coexistence of nulliparity and overweight may amplify each other’s effect on increasing breast cancer risk having strong synergism [161]. Delayed first childbearing proved to be a risk factor of breast cancer among older premenopausal women as well [18, 135], which may also be associated with long term sex hormone imbalance and fertility disorders. High breast cancer risk in correlation with nulliparity and delayed first birth suggests an important role of defective estrogen synthesis and anovulatory disorders in mammary carcinogenesis.

Possible role of fertility medications in the increased risk of breast cancer has been hypothesized. However, large studies were not able to find any associated risk of breast cancer after ovulation provocation and *in vitro* fertilization (IVF) in anovulatory cases [163]. Moreover, an American case-cohort study established that use of clomiphene as treatment for infertility lowers the increased risk of breast cancer in women with ovulatory abnormalities [164]. Recently, overall cancer risk among infertile women before IVF was found to be markedly increased in a large study in Sweden [165]. However, after IVF assisted childbirth, cancer risk was significantly decreased mainly due to a lower than expected risk for breast and cervical cancers. Similarly, a conspicuous great reduction in breast cancer incidence was found among those infertile women who underwent to high dose estrogen treatment as ovulation induction therapy [146]. These recent literary data support the breast cancer lowering value of IVF assisted childbirth among endangered women with anovulatory infertility.

### Correlations Between Postmenopausal Obesity and Breast Cancer Risk in Women 

In postmenopausal estrogen deficient, obese cases the regional distribution of fat deposition near uniformly affects the visceral region in close correlation with their high breast cancer risk [6].

*Obese postmenopausal women never using HRT* are obviously insulin resistant. Moreover, ageing after menopause is a further factor increasing the cancer risk as it is associated with a continuous estrogen loss and parallel advancing insulin resistance [166]. In old obese women, struggle between the harmful sequels of insulin resistance and the low level of protective female sexual steroids will be frequently finished by cancer initiation. These correlations explain the higher breast cancer risk of obese postmenopausal women who do not receive hormone treatment as compared with HRT users.

In *obese HRT user postmenopausal women, *the incidence of breast cancer is equivocally reduced [15]. The protective effect of estrogen substitution may counteract to the obesity associated insulin resistance and their breast cancer risk decreases [19]. In HRT user women the hormonal and metabolic equilibrium becomes similar to that of obese young women with relatively preserved circulatory estrogen levels. Estradiol substitution increases insulin sensitivity [100] yields favorable changes in plasma lipid levels [92] and by its anti-obesity effect decreases the accumulation of visceral fat deposition [167]. All these impacts justify the beneficial anticancer capacity of HRT in obese women after menopause.

A great challenge is to explain the beneficial anticancer impact of HRT in obese postmenopausal women based on the misbelief that they have excessive circulatory estrogen level. To solve this contradiction, some authors assumed that mediators other than estrogen, such as insulin and insulin-like growth factors might confer the obesity associated breast cancer risk after menopause [27].

### Effects of Exogenous Hormone Treatment and Environmental Endocrine Disruptors on Breast Cancer Risk 

* Oral contraceptive (OC) use* replaces the natural menstrual cycle with relatively steady levels and fluctuations of artificial sex hormones.

Recent epidemiological studies have confirmed that combined oral contraceptives provide substantial protection against endometrial and ovarian cancer in the endangered anovulatory women [168, 169]. Effect of OCs on breast cancer risk seems to be controversial. Increased risk was confined to women who have begun pill use as teenagers or those with very long term use [168]. Nevertheless, endometrial, ovarian and breast cancers exhibit conspicuously similar epidemiology and they frequently synchronously appear in anovulatory young women [151, 152]. These observations may suggest that a proper selection of patients and controls would lead to the realistic evaluation of beneficial OC effect even on breast cancer risk.

In PCOS cases, treated with OCs, the volume of cystic ovaries is reduced, ovarian testosterone secretion is decreased and there are favorable effects on carbohydrate and lipid metabolism as well [169]. A recent patent disclosed a method for treating hyperandrogenism and associated conditions, including PCOS by a fatty acid ester of estrogen or an estrogen derivative compound [170]. This therapy seems to be more advantageous against the dangerous dysmetabolism of PCOS cases than OC administration.

*Use of HRT* has highly controversial associations with breast cancer risk. Till now, the carcinogenic capacity of postmenopausal estrogen therapy was a prevailing concept [171]. By contrast, recent literary data support the cancer protective capacity of postmenopausal estrogen treatment both for the moderately and highly hormone responsive organs of women [136, 172]. Results of these studies prophesied a breakthrough regarding the traditional concept of estrogen induced mammary carcinogenesis.

In 2011, the WHI Randomized Controlled Trial strengthened that the estrogen treatment in women with prior hysterectomy resulted in a significantly lower risk for breast cancer than in untreated controls [173]. Breast cancer risk of women with hysterectomy may be near uniformly high because of their abrupt, shocking hormone deprivation. HRT studies on these homogenously selected cases seem to be methodologically fairly strong, yielding unexpectedly correct results [136, 172].

Today, increasing numbers of evidence from the clinical trials suggest that unopposed estrogen as hormone treatment does not increase the risk of breast cancer, and may even reduce it. The earlier concepts regarding the breast cancer risk of hormone replacement therapy are completely changing [174-178].

HRT decreases the accumulation of central fatty tissue in obese postmenopausal women and provides them long term metabolic and health benefits [27, 167]. HRT in older women equivocally attenuates the obesity-associated breast cancer risk [14, 15, 176] similarly to the preserved circulatory estrogen level in case of fat premenopausal women. In conclusion, estrogen treatment in postmenopausal women decreases the obesity and insulin resistance associated breast cancer incidence as well.

Exposure to * environmental estrogens (xenoestrogens)* occurs through diet, household products and cosmetics, but concentrations of single compounds in breast tissue are generally lower than needed for assayable estrogenic responses [179]. Exposure to complex mixtures of estrogenic chemicals in consumer products is regarded as a factor in breast cancer development based on the principle of estrogen induced mammary carcinogenesis.

*Endocrine disruptors (EDs) *are environmental chemicals that are able to bind hormonal receptors. These compounds may disrupt the endocrine regulation of reproduction and adaptive mechanisms and promote the development of endocrine disorders. Since several EDs have a structure similar to that of endogenous steroid hormones such as estrogens, they have an affinity for steroid hormone receptors and may alter the normal hormone-mediated reactions [180]. Environmental EDs may interfere with the development of healthy menstrual cycles in puberty resulting in anovulatory cycles in women.

*Poor natural light exposure* mediates deleterious metabolic and hormonal alterations by excessive melatonin secretion; such as insulin resistance, deficiencies of estrogen, thyroxin and vitamin D [181]. Long term exposition to darkness may deteriorate the development of healthy hormonal equilibrium in puberty endangering the somatic health as well. Light deficiency confers increased breast cancer risk among night shift worker women and dark skinned immigrants in northern countries.

### Estrogen Therapy of Breast Cancer

Before the introduction of antiestrogens, high dose estrogen was successfully administered as endocrine therapy for postmenopausal women with advanced breast cancer. Nowadays, the ambiguous, unreliable therapeutic effects and high toxicity of antiestrogens suggest the necessity of finding new strategies for breast cancer treatment [182]. Recently, the old-fashioned high-dose estrogen treatment in patients with advanced breast cancer and previous estrogen deprivation was demonstrated to be effective in prospective clinical trials. Anti-tumor mechanism of estrogen therapy is under debate, but one can hypothesize that after a long-term antiestrogen treatment estrogen may become an apoptotic trigger rather than a survival signal for breast cancer cells [183].

*Phytoestrogen* containing health supplement compositions are recommended by some patents for the prevention or treatment of postmenopausal breast cancer and for the alleviation of menopausal symptoms [184]. Soybean extract, which has phytoestrogen component proved to be useful as a therapeutic agent for solid cancers, such as breast cancer [185]. A recent patent reported on the use of steroid compounds as anticancer drugs. They have anti-obesity, anti-hyperglycemic, anti-autoimmune and anti-hypercholes-terinemic impacts as well, similarly to estrogen [186].

## CONCLUSION

Obesity-associated hormonal disorders confer breast cancer risk during the whole life of women without any ambiguous interaction between obesity and menopausal status. Erroneous results regarding the breast cancer protective effect of obesity in young women derive mainly from the deceivingly lower tumor incidence among them. Further misleading factor may be that hormonal disorders related to anovulatory infertility are strong cancer risk factors for the breast both in obese and non-obese young women.

* Low incidence rate of breast cancer* in young obese women may be a very important misleading finding. In the majority of premenopausal obese cases a predominantly subcutaneous adipose tissue deposition results in milder insulin resistance being counteracted by their preserved hormonal cycle. Consequently, in obese premenopausal women their circulatory estrogen level confers protective effect against breast cancer rather than obesity.

Tumor cell kinetics may also provide an explanation for the low breast cancer incidence rate in obese premenopausal cases. A long term exposition to harmful environmental and systemic factors is necessary for cancer initiation; moreover the tumor growth from the initiation to clinically diagnosable size takes further several years. This double delay of clinical appearance of breast cancers may frequently result in a postmenopausal tumor, though it was initiated in the premenopausal period.

* Anovulatory infertility and the associated hormonal defects* seem to be stronger breast cancer risk factors than adiposity alone and these alterations are not rare even among control cases with normal weight. Studies on obesity-related breast cancer risk disregarded the random occurrence of these hormonal alterations both among obese cases and lean controls. Moreover, oral contraceptive use is fairly widespread among premenopausal women, which may reverse the mild hormonal defects and attenuates their cancer risk either in obese or in lean cases. Strict selection of healthy, lean control women for obesity associated breast cancer studies and taking into account the OC use among obese cases and controls will justify the health advantage of normal body weight over obesity.

Recognition of the inverse correlation between circulatory estrogen-level and breast cancer risk in obese women should advance our understanding of breast cancer etiology. Moreover, it would promote primary cancer prevention measures and the introduction of causal cancer therapy.

## CURRENT & FUTURE DEVELOPMENTS

Changing concepts concerning breast cancer etiology provide completely new strategies against mammary tumors (Tables **2** and **3**). Circulatory endogenous estrogen was blamed to provoke cancer initiation and promotion in the highly hormone responsive female organs, however, this misbelief yielded fairly controversial results in the clinical and experimental fields of medicine. Nowadays, overwhelming literary data suggest that estrogens have advantageous impact on dysmetabolism and hyperandrogenism, which are essential sources of tumor development.

Recent patents disclose oral contraceptive and metformin derivative treatment possibilities for breast cancer prevention and therapy. Moreover, a new invention provides special estrogen derivatives being metabolically more advantageous for the treatment of PCOS than oral contraceptives. As anovulatory disorders in young women are strong risk factors for breast cancer, insulin resistance screening of asymptomatic occult cases in the population and their hormone treatment would be effective primary breast cancer prevention.

In postmenopausal cases, obesity, metabolic syndrome, type-2 diabetes and hysterectomy are strong risk factors for mammary malignancies. Hormone replacement therapy in these endangered cases would be advantageous preventive measure.

The present practices of breast cancer therapy have fairly controversial results. Worldwide administration of antiestrogen compounds for breast cancer treatment yielded thorough disappointment. Antiestrogens are cytostatic agents blocking the most important regulatory mechanisms associated with estrogen receptors. They do not have only toxic effects but also strong carcinogenic capacity. Recently, scientists in some centers confess, that one armed estrogen treatment is rather cancer protective than carcinogenic. Certain patents disclose the beneficial effects of phytoestrogens in breast cancer prevention and therapy. Moreover, publications on the high dose estrogen treatment of advanced breast cancer cases are regularly returning and the results are fairly encouraging. Today, we are on the route to regard estrogens as beneficial anti-cancer drugs rather than carcinogenic agents.

## Figures and Tables

**Table 1.** Lifelong Changes in the Hormonal Status of Obese Women and their Breast Cancer Risk.

Life Periods of Obese Women	Estrogen Level	Insulin Resistance	Breast Cancer Risk
Children	↓	↑	↑
Adolescent girls	↓↓	↑↑	↑↑
Premenopausal women			
Type-2 diabetes	↓	↑	↑
Anovulation	↓	↑	↑
Nulliparity	↓	↑	↑
Contraceptive use	↑	↓	↓
Multiparity	↑	↓	↓
*In vitro* fertililization	↑	↓	↓
Postmenopausal women			
HRT user	↑	↓	↓
Non HRT user	↓	↑	↑
Type-2 diabetes	↓↓	↑↑	↑↑
Hysterectomy	↓↓	↑↑	↑↑

**Table 2.** Hormonal Risk Factors for Breast Cancer.

Traditional Concept	Author’s New Concept
Excessive circulatory estrogen level	Reduced circulatory estrogen level
Increased aromatase activity	Defective aromatase activity
Unopposed estrogen level	---------
Ovulatory peak of estrogen level	Anovulatory disorder
Contraceptive use	Defective hormonal cycle
Cross talk of estrogen and GFs	Missing surveillance of estrogen on GFs
Xenoestrogens	---------
Hormone replacement therapy	Missing hormone replacement therapy
Excessive light exposure	Light deficiency
**Androgen excess**	**Androgen excess**
**Hyperinsulinemia**	**Hyperinsulinemia**

**Table 3.** Protective Hormonal Factors Against Breast Cancer.

Traditional Concept	Author’s New Concept
Obesity (in young)	Normal weight
Anovulation	Healthy ovulatory cycles
Hysterectomy (preventive)	Preservation of gynecological organs
Antiestrogen (preventive)	Estrogen prevention
